# Microplastics and nanoplastics as potential ecological oncogenic co-drivers: rewiring host–microbe interactions in cancer

**DOI:** 10.3389/fcimb.2026.1923626

**Published:** 2026-10-06

**Authors:** Richard Ponce-Cusi, Claudia Barletta-Carrillo, Nesstor Pilco-Ferreto

**Affiliations:** 1 Escuela Profesional de Medicina, Facultad de Ciencias de la Salud, Universidad Nacional de Moquegua, Moquegua, Peru; 2 Laboratorio de Genética Humana, Facultad de Ciencias Biológicas, Universidad Nacional Mayor de San Marcos, Lima, Peru

**Keywords:** carcinogenesis, host–microbe interactions, immune evasion, microbiome, microplastics, nanoplastics, tumor microenvironment

## Abstract

Microplastics and nanoplastics (MPs/NPs) are increasingly recognized as biologically active interfaces that may reshape host–microbe interactions in carcinogenesis. This review proposes a hypothesis-generating mechanistic framework in which MPs/NPs may influence cancer-related processes through the integration of environmental exposure, epithelial barrier disruption, immune dysregulation, and tumor microenvironment remodeling. Although applicable to multiple epithelial interfaces, the framework primarily focuses on the intestinal mucosa, where the most substantial evidence exists. Through plastisphere formation, MPs/NPs adsorb pollutants, virulence factors, and microbial communities, potentially facilitating pathogen persistence, horizontal gene transfer, and antibiotic resistance enrichment. Upon host exposure, MPs/NPs have been associated with compromised epithelial integrity, enhanced microbial translocation, and activation of inflammatory pathways involving pattern recognition receptors, NF-κB, and the NLRP3 inflammasome. At the microbiome level, MPs/NPs may promote dysbiosis, alter microbial metabolism, and favor pro-inflammatory and genotoxic metabolites that could reinforce oncogenic signaling. These processes may converge to sustain chronic inflammation, immune evasion, extracellular matrix remodeling, angiogenesis, and tumor-permissive niches. While experimental and biomarker-based evidence support this axis, direct human data remain limited. Collectively, this review provides a conceptual basis for understanding environmentally associated carcinogenesis through MPs/NPs as ecological mediators bridging the environmental exposures, microbiome, and cancer biology.

## 1 Introduction

Microplastics and nanoplastics (MPs/NPs) have transitioned from being considered inert environmental debris to recognized pervasive contaminants across terrestrial and aquatic ecosystems. While definitions vary across regulatory and research frameworks, microplastics are generally defined as synthetic polymer particles ranging from 1 μm to 5 mm, whereas nanoplastics are typically defined as particles smaller than 1 μm (Schwabl et al., 2019; Vethaak and Legler, 2021). For practical purposes, these particles are often collectively referred to as MPs/NPs, a convention adopted throughout this review. Their widespread distribution enables incorporation into food webs and facilitates accumulation across multiple biological compartments, with their presence documented in diverse organ systems in both experimental models and human tissues. These particles originate from the progressive fragmentation of larger plastic debris or are directly released as primary MPs/NPs, such as microbeads and synthetic fibers, thereby representing a persistent and structurally heterogeneous class of environmental stressors characterized by high durability, size-dependent penetrability, and reactive surface properties (Wright and Kelly, 2017). Notably, the physicochemical and biological behavior of MPs/NPs varies considerably depending on polymer type, such as polyethylene (PE), polystyrene (PS), polypropylene (PP), polyvinyl chloride (PVC), polyethylene terephthalate (PET), and high-density polyethylene (HDPE), morphology, surface chemistry, and the presence of adsorbed contaminants or microbial biofilms. While most experimental studies rely on commercially available pristine particles, environmental MPs/NPs undergo weathering processes that modify their surface properties and biological reactivity, a distinction relevant when interpreting mechanistic evidence (Prata, 2023; Ponce-Cusi et al., 2025; Patra et al., 2026).

Importantly, MPs/NPs should be regarded as dynamic bioactive interfaces capable of mediating complex physicochemical and biological interactions. Their hydrophobic surfaces facilitate the adsorption and concentration of heavy metals (Ashton et al., 2010), persistent organic pollutants (Mato et al., 2001), and diverse microbial communities, collectively referred to as the plastisphere, which is considered a distinct and emergent ecological niche associated with synthetic polymers (Zettler et al., 2013; Wu et al., 2025b). This interface functions as a reservoir of toxic compounds and a selective microenvironment that reshapes microbial composition, often favoring opportunistic pathogens and antimicrobial-resistant taxa (Wu et al., 2019).

Through these surface-mediated interactions, MPs/NPs acquire the capacity to directly engage with host biological systems following exposure. Upon ingestion or inhalation, they accumulate across multiple compartments, including the gastrointestinal tract, lungs (Amato-Lourenço et al., 2021; Jenner et al., 2022), bloodstream, and lymphoid organs, where they induce oxidative stress, disrupt mitochondrial function, compromise tight junction integrity, and initiate both local and systemic inflammatory cascades, thereby exacerbating immune activation, apoptosis, and necrosis in animal models (Zhang et al., 2023b).

MPs/NPs establish novel ecological interfaces, thereby promoting microbial persistence (Sooriyakumar et al., 2022) and intensifying host exposure (Wright and Kelly, 2017; Zhang et al., 2023b). In contrast, sustained exposure to MPs/NPs-associated microbial signals may also contribute to immune dysregulation and evasion, altering host defense mechanisms and enabling persistent host–pathogen interactions (Zhang et al., 2023b; Cheng et al., 2024; Fan and Ha, 2025). These processes cumulatively establish a mechanistic link between environmental exposure, microbial dynamics, and immune imbalance, factors underlying chronic inflammation and cancer progression (**Figure 1**).

**Figure 1:**
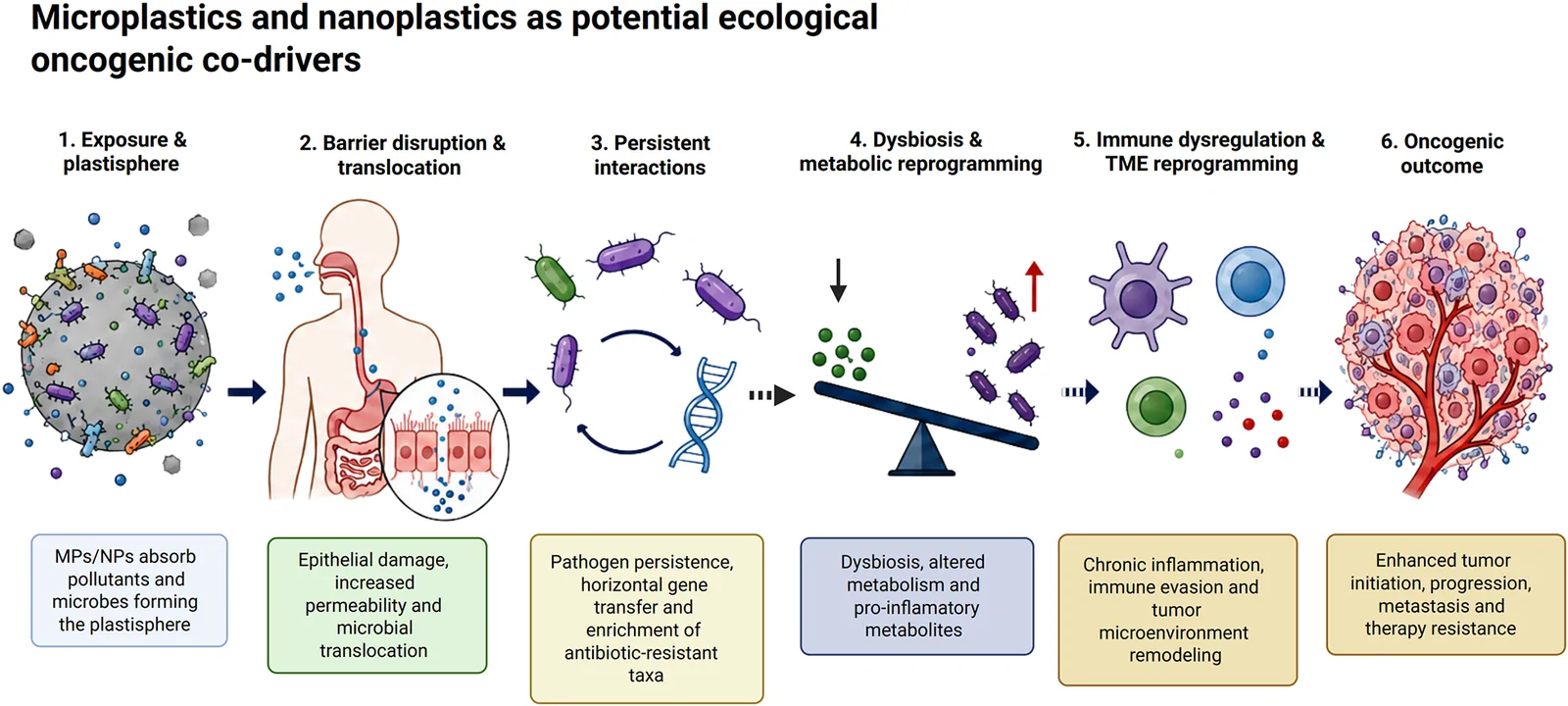
Proposed mechanistic framework by which MPs/NPs act as ecological oncogenic co-drivers. Following environmental exposure, MPs/NPs adsorb pollutants and microbial communities, giving rise to the plastisphere as a mobile ecological niche. Upon host exposure, these particles disrupt epithelial barriers, increase permeability, and facilitate microbial translocation. Persistent host–pathogen interactions then promote pathogen survival, horizontal gene transfer, and enrichment of antibiotic-resistant taxa. At the microbiome level, MPs/NPs drive dysbiosis, metabolic imbalance, and the accumulation of pro-inflammatory metabolites. These processes converge on chronic inflammation, immune dysregulation, and tumor microenvironment reprogramming, ultimately favoring oncogenic signaling, tumor initiation, progression, metastasis, and therapy resistance. Overall, the image illustrates how MPs/NPs integrate the exposome, microbiome, and host biology into a unified ecological pathway of carcinogenesis. (Created with BioRender.com).

This review proposes a mechanistic framework positioning MPs/NPs as biologically active ecological drivers that disrupt host–pathogen homeostasis, modulate immune evasion and epithelial dysfunction, and reprogram the tumor microenvironment to promote cancer initiation and progression. For this purpose, this review was conducted as a narrative, hypothesis-generating synthesis designed to integrate current evidence on the interactions between MPs/NPs, host–microbe dynamics, immune dysregulation, and cancer-related mechanisms. This review was not intended as a systematic review or meta-analysis. Literature was identified through searches of PubMed/MEDLINE, Scopus, and Web of Science. Search terms included combinations of microplastics, nanoplastics, plastisphere, microbiome, host–microbe interaction, host–pathogen interaction, dysbiosis, immune dysregulation, inflammation, tumor microenvironment, and carcinogenesis. The search covered publications from January 2005 to March 2026, and the final search was performed on March 30, 2026.

Priority was given to original experimental studies investigating MPs/NPs exposure, host–microbe interactions, epithelial barrier dysfunction, immune mechanisms, microbiome alterations, and cancer-related biological processes. Human studies were preferentially considered whenever available, whereas animal and *in vitro* studies were incorporated to support mechanistic interpretation in areas with limited clinical evidence. Recent high-quality review articles were used to provide conceptual context and identify emerging themes. As the objective was to develop an integrative mechanistic framework rather than perform quantitative evidence synthesis, no formal risk-of-bias assessment or systematic study selection process was undertaken.

### 1.1 Cancer as an ecological disease in disrupted host–microbe–environment systems

A central determinant within this ecological landscape is the triad between host epithelial integrity, immune surveillance, and microbial composition. Mucosal surfaces of the gastrointestinal and respiratory tracts, as well as the skin, function as dynamic interfaces where host–microbe interactions are tightly regulated. Disruption of this equilibrium promotes dysbiosis, characterized by pathobiont overgrowth, loss of microbial diversity, and increased production of inflammatory and genotoxic metabolites (Garrett, 2015; Sepich-Poore et al., 2021). These alterations impair immune tolerance, sustain chronic low-grade inflammation, and create microenvironments conducive to neoplastic transformation. This is exemplified by *Fusobacterium nucleatum* and *Escherichia coli*, which can colonize colorectal tumors and modulate immune evasion through adhesins (Rubinstein et al., 2013), genotoxins (Arthur et al., 2012), and immunomodulatory factors (Kostic et al., 2013).

Within this framework, carcinogenesis can be understood as the collapse of a tripartite homeostatic network linking environmental exposures, microbial ecosystems, and host defenses. MPs/NPs contribute to this disruption by acting as dynamic carriers of microbial communities, chemical contaminants, and resistance-associated elements, while their physicochemical properties enable the adsorption and concentration of biofilms, virulence factors, and pollutants. In doing so, they destabilize host–microbe symbiosis, amplify pathological crosstalk between microbial communities and host tissues, and reinforce conditions of epithelial dysfunction, immune dysregulation, and persistent inflammation (Pan et al., 2024; Li et al., 2025b).

Tumorigenesis therefore emerges from the convergence of environmental exposures, microbial imbalance, and host immune responses (Lin et al., 2024; Albukhari, 2025; Wen and Lin, 2025). From this perspective, MPs/NPs may act as ecological cofactors linking environmental exposure with microbial ecosystem perturbation, largely through plastisphere-associated processes.

### 1.2 Scope and rationale: connecting MPs/NPs to host–microbe–cancer dynamics

MPs/NPs exert profound biological effects at epithelial interfaces, where they compromise barrier integrity by disrupting tight junction architecture and increasing mucosal permeability, thereby driving the translocation of microorganisms and microbial-derived products into host tissues (Jin et al., 2019). This establishes a critical mechanistic interface between environmental exposure and host–microbe interactions, further amplified by MPs/NPs-associated microbial communities that trigger pattern recognition receptors (PRRs) and inflammasome pathways, particularly Toll-like receptors (Alijagic et al., 2023; Lin et al., 2024), culminating in systemic inflammation and progressive immune dysregulation (Fan and Ha, 2025).

Altogether, these processes reshape the local microenvironment by altering microbial metabolism, modulating immune function, and activating oncogenic signaling pathways that promote malignant transformation and tumor progression (Arthur et al., 2012; Sepich-Poore et al., 2021). This framework is especially relevant in epithelial cancers arising at high-exposure interfaces, such as the intestine, lung, and liver, where continuous interplay among MPs/NPs, microbial communities, and host barriers fosters chronic inflammation, immune evasion, and oncogenesis (Sepich-Poore et al., 2021; Ponce-Cusi et al., 2025).

## 2 MPs/NPs as physical and chemical vectors of pathogens

### 2.1 Adsorption and transport of microbial biofilms on MPs/NPs surfaces

Upon release into the environment, MPs/NPs rapidly acquire a biological identity through the development of surface-associated microbial biofilms, a phenomenon that underpins the concept of the plastisphere (Zettler et al., 2013; Mohamed et al., 2024). This ecological niche supports diverse microbial consortia, including commensal, opportunistic, and pathogenic microorganisms embedded within a self-produced extracellular polymeric substance (EPS) matrix. This matrix promotes adhesion, surface colonization, and pollutant sequestration, thereby conferring protection and metabolic advantages to resident microbiota (Flemming and Wingender, 2010; Kesy et al., 2016).

The physicochemical properties of MPs/NPs, particularly hydrophobicity, surface roughness, and polymer composition (Yang et al., 2023b), modulate the selective enrichment of distinct microbial taxa and virulence profiles (Kirstein et al., 2018). For example, PE and PS particles harbor microbial communities that differ from those found on natural substrates, with biofilm-associated traits that enhance pathogenic potential (Kirstein et al., 2018; Wu et al., 2025b).

Importantly, MPs/NPs act as mobile microbial reservoirs, enabling long-distance dispersal of biofilm-embedded bacteria (Wu et al., 2019; Wei et al., 2024) and viruses across ecosystems (Tang et al., 2024; Wang et al., 2024; Capuano et al., 2026). This transport facilitates the seeding of distant niches and increases the likelihood that pathogenic taxa will enter human-associated environments, including water supplies (Li et al., 2018; Wu et al., 2019; Ibrahim et al., 2025), food chains (Cox et al., 2019), and indoor air (Amato-Lourenço et al., 2021; Jenner et al., 2022). This mechanism (**Table 1**, mechanism a) is supported by direct evidence of viable pathogen transport on MPs/NPs surfaces in aquatic and wastewater systems (He et al., 2018; Wu et al., 2019; Lai et al., 2022), although the extent to which such transport translates to human exposure remains an area of active investigation.

**Table 1:** Distinct mechanisms by which MPs/NPs may influence host–microbe interactions and cancer-related processes.

Mechanism	MPs/NPs	Size	Model/Host	Key findings	Evidence level	References
(a) Transport of viable environmental pathogens	PE	100 μm	Aquatic biofilm	Selective enrichment of *Pseudomonas* spp. on plastic surfaces.	Direct (iii)	(Wu et al., 2019)
	PE, PP	N/A	Seawater	Detection of potentially pathogenic *Vibrio* spp. on plastic particles.	Direct (iii)	(Kirstein et al., 2016)
	PE	MPs	Wastewater	Rapid colonization by opportunistic pathogens (*Arcobacter*, *Aeromonas*, *Vibrio*).	Direct (iii)	(Lai et al., 2022)
(b) Transport of microbial products (LPS)	PS	NPs	Immune cells	MPs/NPs act as scaffolds for LPS and modify host immune recognition.	Direct (ii)	(Yang et al., 2023a)
	PS	MPs	*In vitro*/*in vivo*	MPs/NPs increase cellular sensitivity to inflammasome activation.	Direct (ii)	(Ying et al., 2024)
(c) Direct particle-induced epithelial injury	PS	50 nm/5 μm	Intestinal epithelial cells	Ferroptosis (NPs) vs. metabolic reprogramming (MPs); both disrupt barrier function.	Direct (ii)	(Cheng et al., 2025)
	PS	100 nm/5 μm	Intestinal epithelial cells	ROS-mediated epithelial cell death and barrier dysfunction.	Direct (ii)	(Liang et al., 2021)
	PS	1–10 μm	Mouse (inhalation)	Pulmonary epithelial injury, inflammation, and tissue remodeling	Direct (ii)	(Lu et al., 2021)
	PS	NPs	Skin cells	Mitochondrial oxidative stress, AIM2 inflammasome activation, local inflammation.	Direct (ii)	(Han et al., 2024)
(d) Translocation of endogenous microbiota	N/A	N/A	Barrier biology review	Translocation of commensal/pathobiont bacteria sustains systemic immune activation.	Indirect (iv/v)	(Fine et al., 2020)
	PS	70 nm/5 μm	Mouse	Barrier disruption and dysbiosis facilitate inflammation.	Indirect (iv/v)	(Qiao et al., 2021)
(e) Independent effects of adsorbed chemicals	PS	NPs	*In vitro*/*in vivo*	Co-transported xenobiotics increase carcinogenic risk and oxidative stress.	Direct (ii)	(Sun et al., 2023)
	PS + DEHP	MPs	Mouse	ER stress (GRP78/CHOP/Bcl-2) and apoptosis induced by co-exposure.	Direct (ii)	(Wang et al., 2022)

Evidence categories: (i) direct co-exposure studies (MPs/NPs + microorganisms + host); (ii) studies of MPs/NPs and hosts without a microbial component; (iii) studies of the plastisphere or microbial ecology without a host component; (iv) studies of cancer-associated microorganisms without plastic exposure; (v) mechanistic analogies or hypotheses.

From an epidemiological perspective, these plastisphere-associated biofilms are particularly concerning because they display increased antimicrobial tolerance, driven by restricted antibiotic diffusion, localized microenvironmental changes (Zhang et al., 2025a), and accelerated horizontal gene transfer within the EPS matrix (Arias-Andres et al., 2018). Consequently, MPs/NPs function not only as transport platforms but also as evolutionary incubators that enhance the persistence, amplification, and dissemination of virulence determinants and antimicrobial resistance genes (Zhang et al., 2025a).

It is worth emphasizing, however, that the plastisphere characterized in environmental settings differs fundamentally from the particle surface that ultimately interfaces with host tissues. This distinction arises because the composition and viability of surface-associated microbial communities may be substantially remodeled upon exposure to gastrointestinal, pulmonary, or systemic fluids (**Figure 2**).

**Figure 2:**
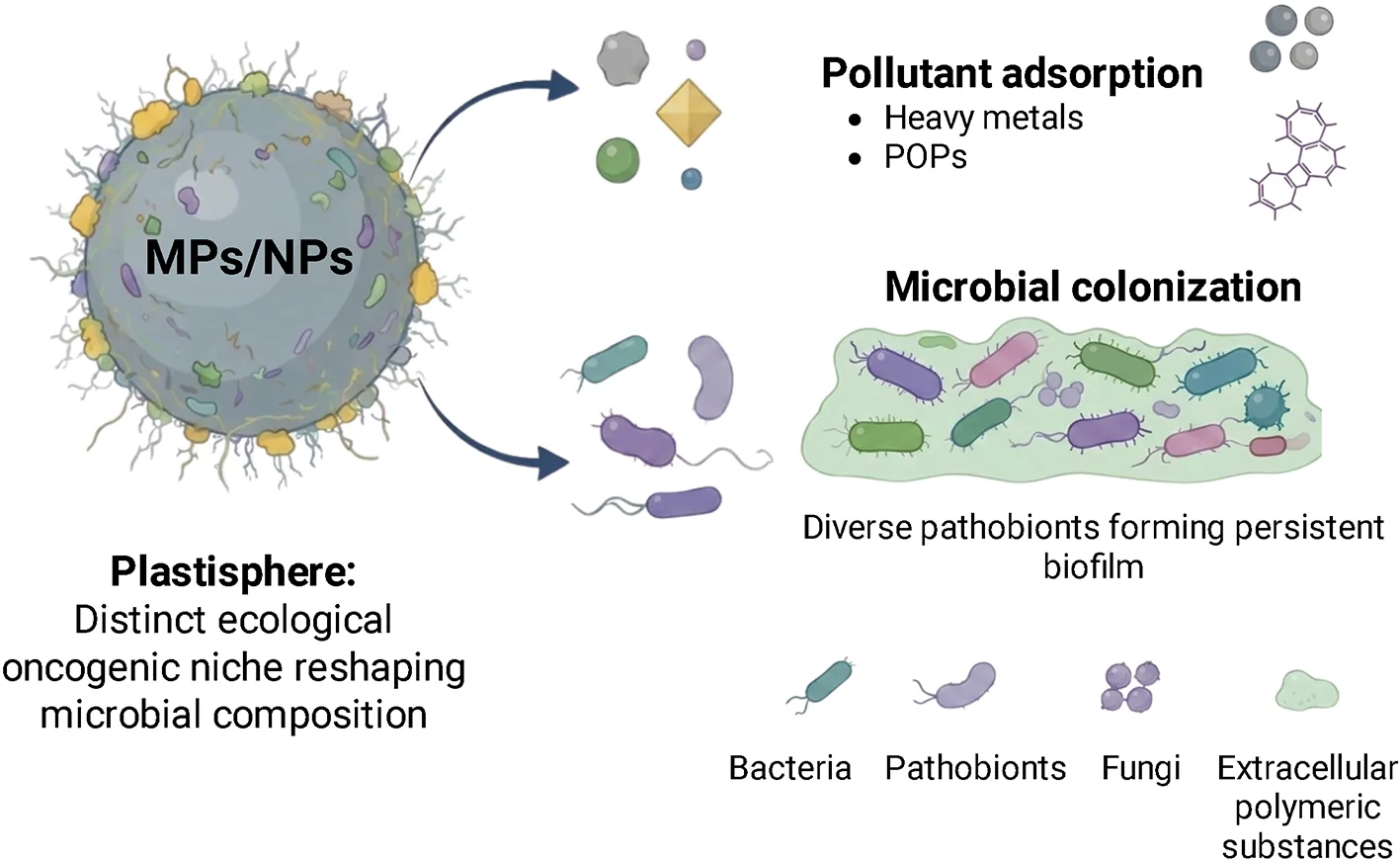
The plastisphere as a mobile ecological driver of host–microbe interface and oncogenic risk. Through plastisphere formation, MPs/NPs create a distinct ecological niche that supports the persistence of diverse microbial communities, including bacteria, pathobionts, fungi, and extracellular polymeric substances organized within biofilm-like structures. By concentrating toxicants and reshaping microbial composition, the plastisphere functions as a mobile interface that enhances pathogen persistence, favors high-risk microbial assemblages, and contributes to environmentally relevant host exposure with potential oncogenic implications. (Created with BioRender.com).

### 2.2 Plastic-associated bacteria and virulence traits: the case of *Escherichia coli pks*+

A prominent example of plastic-associated microbial virulence is provided by *Escherichia coli* strains harboring the *pks*+ genomic island, a biosynthetic gene cluster responsible for the production of colibactin, a genotoxic secondary metabolite with potent DNA-alkylating activity. Colibactin induces DNA interstrand crosslinks, double-strand breaks, and chromosomal instability in host cells, thereby promoting genomic instability, cellular senescence, and inflammatory responses that favor colorectal tumorigenesis (Nougayrède et al., 2006; Pleguezuelos-Manzano et al., 2020).

The virulence potential of *E. coli pks+* is markedly amplified when these strains are incorporated into biofilms formed on MPs/NPs surfaces. Owing to their hydrophobicity and surface roughness, MPs/NPs facilitate initial microbial adhesion and protect embedded cells from environmental fluctuations and host-derived immune stress. Experimental evidence shows that *E. coli* grown on MPs/NPs exhibits enhanced biofilm formation and greater tolerance to antibiotics and oxidative stress than planktonic bacteria or those growing on inert substrates such as glass (Gross et al., 2025; Li et al., 2025a). This example illustrates mechanism (a) in **Table 1** and also involves microbial product delivery (mechanism b) through the genotoxic activity of colibactin.

This interaction has direct oncological relevance. *E. coli pks+* has been consistently detected in the intestinal mucosa of patients with colorectal cancer, where it interacts with the tumor microenvironment. Colibactin-producing strains induce epithelial senescence and a senescence-associated secretory phenotype that amplifies pro-inflammatory and pro-tumorigenic signaling (Cougnoux et al., 2014), while experimental models show accelerated adenoma formation, particularly under conditions of epithelial injury and microbiota imbalance (Cuevas-Ramos et al., 2010; Arthur et al., 2012). Together, these findings support the view that MPs/NPs can enhance the persistence, spread, and oncogenic potential of high-risk pathobionts within host-associated environments.

### 2.3 Plastic particles as shelters and vehicles for antibiotic-resistant pathogens

#### 2.3.1 MPs/NPs as selective ecological niches for AMR enrichment and horizontal gene transfer

The global escalation of antimicrobial resistance (AMR) has highlighted environmental reservoirs as critical nexuses for the persistence and dissemination of resistance determinants. Among these, MPs/NPs have emerged as novel ecological niches that selectively concentrate antibiotic-resistant bacteria (ARB) and antimicrobial resistance genes (ARGs) through plastisphere-associated biofilm dynamics (Chaukura et al., 2021; Zhang et al., 2025a). These particles function as synthetic islands where microbial communities interact under selective pressure imposed by plastic-bound pollutants, including antibiotics, heavy metals, and biocides, thereby favoring the mobilization of resistance elements through plasmids, integrons, and transposons (Ding et al., 2022; Qi et al., 2024; Siddique et al., 2025).

Within these biofilms, EPS establish diffusion barriers and redox gradients that protect resident bacteria from environmental stressors and antimicrobial agents while enhancing survival and facilitating horizontal gene transfer (HGT), particularly through conjugation (Li et al., 2024b; Zhou et al., 2024). In aquatic systems, plastisphere communities consistently exhibit higher ARG abundance and diversity than free-living or naturally attached microbial populations (Yang et al., 2019; Witsø et al., 2025) and the biofilm-associated lifestyle on plastic surfaces further promotes the dissemination of antimicrobial resistance genes and mobile genetic elements (Arias-Andres et al., 2018; Zhou et al., 2024). PE, PP, and PS particles are especially prone to colonization by multidrug-resistant taxa such as *Vibrio, Pseudomonas, and Enterobacteriaceae* (Kirstein et al., 2016; Wu et al., 2019; Metcalf et al., 2024). These biofilms also display transcriptional signatures associated with enhanced horizontal gene transfer and biofilm maturation, conferring heightened tolerance to antibiotics and disinfection protocols (Zhou et al., 2024; Zhang et al., 2025a). Importantly, resistance genes such as beta-lactam, aminoglycoside, and tetracycline resistance determinants have been repeatedly detected in MPs/NPs-associated biofilms, with evidence of transfer between environmental and clinically relevant strains (Joannard and Sanchez-Cid, 2024; Witsø et al., 2025; Liu et al., 2026a). Experimental studies further show that MPs/NPs prolong ARB persistence under sub-inhibitory antibiotic concentrations, and plastic-associated biofilms can accelerate ARG dissemination relative to planktonic environments (Arias-Andres et al., 2018; Gross et al., 2025).

#### 2.3.2 MPs/NPs as vectors linking environmental resistomes to human and oncological risk

The environmental resistome comprises the collective reservoir of ARGs across natural, engineered, and host-associated microbial communities, including both pathogenic and commensal organisms. In the context of MPs/NPs exposure, this resistome should be understood not merely as an antimicrobial resistance framework, but as a dynamic ecological system in which resistance, virulence, persistence, and stress adaptation converge. MPs/NPs-associated resistomes may contribute to microbial persistence, antimicrobial resistance, and ecological changes with potential relevance to host exposure and chronic inflammatory processes (Joannard and Sanchez-Cid, 2024; Witsø et al., 2025).

This ecological convergence has direct implications for human health. MPs/NPs have been detected in seafood, salt, drinking water, and indoor air, where they act as vectors for viable microbial communities capable of colonizing mucosal surfaces or entering systemic circulation through epithelial translocation (Wright and Kelly, 2017; Cox et al., 2019). Once internalized, these resistant and potentially pathogenic communities may disrupt intestinal microbial homeostasis, promote dysbiosis, and sustain chronic inflammation (Sepich-Poore et al., 2021; Yee et al., 2021). These risks may be particularly relevant in immunocompromised settings, including patients with mucosal barrier dysfunction or those receiving chemotherapy, where exposure to MPs/NPs-associated antibiotic-resistant bacteria could plausibly increase susceptibility to opportunistic infection and compromise antimicrobial effectiveness. In this context, the documented convergence of MPs, antibiotics, ARB, and ARGs across environmental and wastewater interfaces (Yang et al., 2024) supports the view that MPs/NPs can act as ecological carriers of clinically relevant resistant microbial assemblages, which raises concerns about downstream impacts on vulnerable hosts (Yee et al., 2021; Syranidou and Kalogerakis, 2022).

Taken together, these findings suggest that MPs/NPs may function as active ecological interfaces linking environmental pollution, antimicrobial resistance, and oncogenic risk. Their persistence across ecosystems and ability to connect environmental and clinical microbiomes are consistent with a model in which MPs/NPs could amplify microbial dissemination, maintain resistance traits, and facilitate interspecies gene flow, thereby potentially increasing the likelihood that chronic inflammation, immune dysregulation, and infection susceptibility converge in cancer-relevant settings (**Table 2**).

**Table 2:** MPs/NPs reshape host–pathogen interactions across biological and ecological scales.

Dimension	Infection without MPs/NPs	Infection with MPs/NPs	Experimental/analytical implications	References
Effective Pathogen Load	Determined by direct exposure (water, air, fomites)	Increased via adsorption and concentration on MPs/NPs surfaces	Quantification of CFU or viral load per volume vs per MPs/NPs particle.	(Tang et al., 2024; Ibrahim et al., 2025)
Mode of Transport	Free diffusion or classical biological vectors	Passive transport via MPs/NPs with enhanced stability and long-distance dispersion	MPs/NPs exhibit high environmental mobility, facilitating their widespread dispersal, and microbial transport.	(He et al., 2018; Wei et al., 2024)
Pathogen Survival	Limited by environmental stressors (UV, pH, desiccation)	Enhanced persistence within plastisphere-associated biofilms	Viability assays (time-kill curves).	(Zettler et al., 2013; Mohamed et al., 2024)
Microbial Evolution and Phenotypic Selection	Baseline microbial traits with limited environmental selection pressure	MPs/NPs select for biofilm-forming, multidrug-resistant phenotypes with enhanced persistence and post-exposure adaptation	Confocal laser scanning microscopy with metagenomics (16S rRNA/shotgun sequencing).	(Gross et al., 2025)
Epithelial Barrier Disruption and Exposome Integration	Intact epithelial barrier with controlled host–microbe interactions	MPs/NPs as part of the exposome disrupt epithelial integrity, promoting dysbiosis, microbial translocation, and chronic inflammation	Barrier integrity assays, microbiome diversity analysis, translocation models, cytokine profiling.	(Losol et al., 2023)
Translocation Mechanisms	Limited pathogen entry via regulated epithelial transport	MPs/NPs enable translocation via barrier disruption, facilitating systemic dissemination.	Intestinal models, permeability assays, tracer studies, microbiome/metabolite analysis.	(Shu et al., 2023)
MPs/NPs-Mediated Immunogenicity Modulation and Innate Immune Mimicry	Immune response determined by intrinsic microbial PAMPs	MPs/NPs alter microbial and molecular structure, modifying immune recognition and inflammatory responses. MPs/NPs mimic pathogen-associated signals.	PRR activation assays, cytokine profiling, host–pathogen interaction models and TLR signaling analysis.	(Park et al., 2023; Yang et al., 2023a)
Microbiome Dysbiosis and Systemic Metabolic Reprogramming	Balanced microbiome with stable SCFA production and immune homeostasis	MPs/NPs induce dysbiosis characterized by loss of SCFA-producing bacteria, enrichment of pro-inflammatory taxa, and systemic metabolic and inflammatory alterations	Microbiome sequencing, metabolomics SCFAs, pathway analysis, host–microbiome interaction studies.	(Bora et al., 2024; Yang et al., 2026)
Environmental and Anthropogenic Reservoirs of High-Risk Microbial Communities	Pathogens distributed across environmental matrices with limited structured enrichment	MPs/NPs generated in natural and engineered systems act as reservoirs and amplifiers of pathogenic communities, enriching virulence traits and ARGs.	Environmental sampling, water system modeling, microbial network analysis, infection assays, metagenomics.	(Metcalf et al., 2024; Yang et al., 2024)
Combined Toxicity	Natural microbial equilibrium shaped by environmental factors	MPs/NPs type- and dose-dependent selective pressure reshaping microbial diversity and enriching pathogenic taxa	Microcosm experiments, microbial diversity, metagenomics.	(Qi et al., 2024)
Antimicrobial Resistance	Baseline or induced resistance	Enrichment within plastisphere ARG reservoirs	qPCR and resistome profiling.	(Yang et al., 2019; Witsø et al., 2025).

## 3 Epithelial barrier disruption and pathogen translocation

MPs/NPs are increasingly recognized as important disruptors of epithelial barrier integrity, establishing a critical mechanistic interface between environmental exposure and host exposure to microorganisms and microbial-derived products (Losol et al., 2023). Rather than acting solely as inert toxicants, MPs/NPs can function as bioactive interfaces that induce oxidative stress, inflammation, and structural damage across epithelial systems (Dong et al., 2020; Bishop et al., 2025), thereby facilitating prolonged exposure to microbial-derived stimuli (Liang et al., 2021). See **Figure 3**.

**Figure 3:**
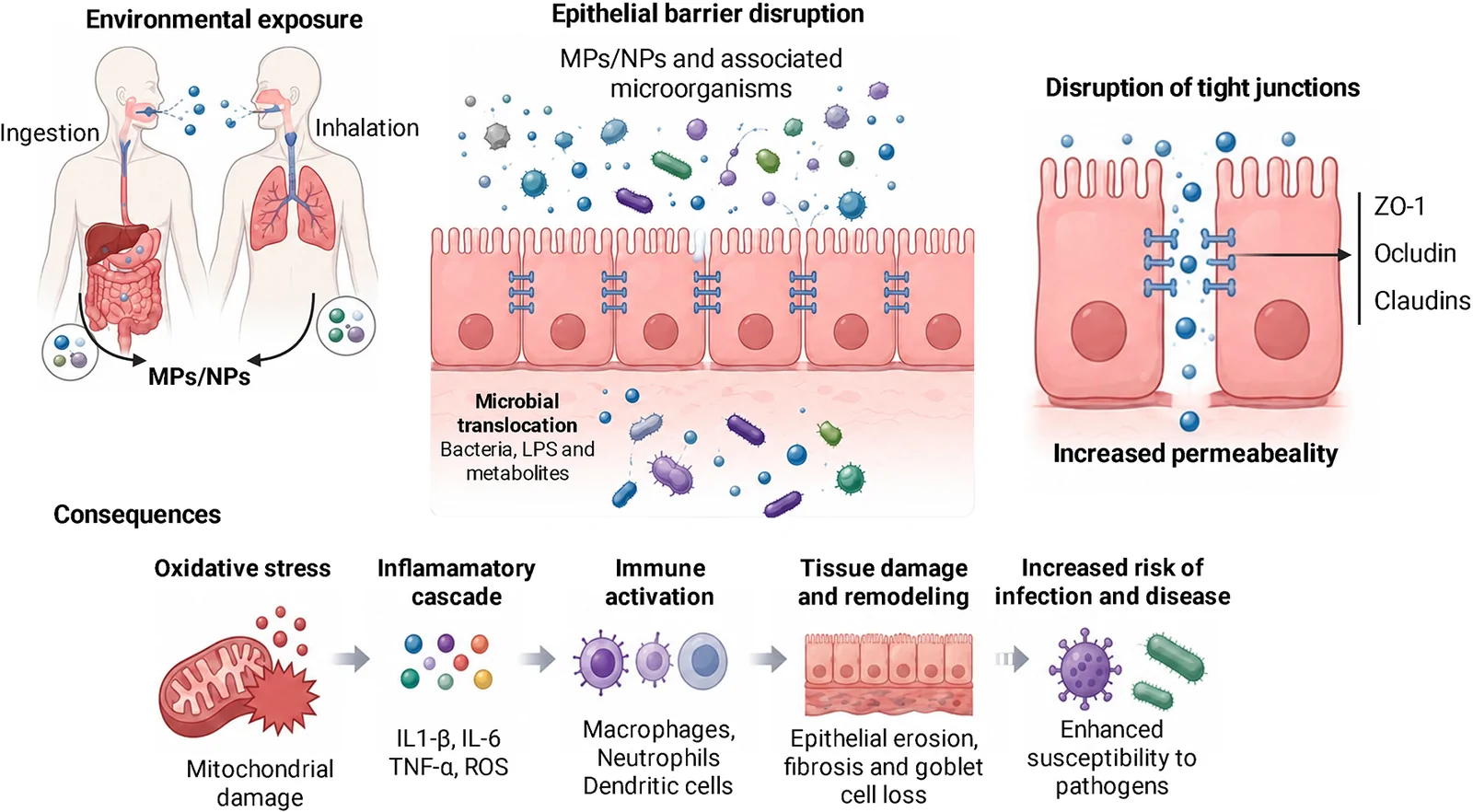
Environmental exposure to MPs/NPs and epithelial barrier disruption: a gateway to microbial translocation and inflammation. Following host exposure, MPs/NPs and associated microorganisms interact with mucosal surfaces, disrupt tight junction proteins including ZO-1, occludin, and claudins, and increase epithelial permeability. This barrier dysfunction facilitates the translocation of bacteria, lipopolysaccharide (LPS), and microbial metabolites across the epithelium. Downstream consequences include mitochondrial damage and oxidative stress, activation of inflammatory cascades involving cytokines such as IL-1β, IL-6, TNF-β, and ROS, recruitment of immune cells including macrophages, neutrophils, and dendritic cells, and progressive tissue damage characterized by epithelial erosion, fibrosis, and goblet cell loss. Collectively, these changes increase susceptibility to infection and establish a mechanistic link between MPs/NPs exposure, host–pathogen interactions, and chronic inflammatory disease processes. (Created with BioRender.com).

### 3.1 Mechanisms of epithelial barrier disruption

Across intestinal and pulmonary epithelia, MPs/NPs impair barrier integrity through convergent mechanisms centered on oxidative stress, inflammatory signaling, epithelial cell death, and disruption of junctional architecture (Dong et al., 2020). In the intestine, PP MPs/NPs increase epithelial permeability, reduce mucus secretion, and downregulate tight junction proteins including ZO-1, occludin, and claudin-1, while simultaneously activating ROS-dependent inflammatory pathways such as NF-κB/NLRP3/IL-1β/MLCK, thereby promoting barrier dysfunction (Jia et al., 2023; Zeng et al., 2024). Size-dependent evidence further indicates that intestinal injury may arise through distinct mechanisms, with smaller particles favoring intracellular oxidative injury and ferroptotic damage, whereas larger particles promote epithelial disruption through mechanical stress and metabolic reprogramming (Cheng et al., 2025). These findings correspond to mechanism (c) in **Table 1**, direct particle-induced epithelial injury, which is supported by extensive *in vitro* and animal evidence.

In pulmonary tissues, inhaled PS similarly induces ROS-dependent epithelial injury, inflammatory infiltration, apoptosis, and fibrosis, while decreasing barrier function and activating Toll-like receptors (TLR) 2/NF-κB-associated inflammatory signaling (Lu et al., 2021; Cao et al., 2023a). Whole-body inhalation studies further show that respiratory exposure disrupts pulmonary oxidative balance, impairs antioxidant defenses, and promotes inflammation and tissue remodeling, with evidence of subsequent intertissue transport beyond the lung (Chen et al., 2025). In summary, these findings support the concept that MPs/NPs compromise epithelial integrity through shared but tissue-contextualized mechanisms, thereby increasing permeability, weakening mucosal defense, and facilitating the entry of environmental agents and microbial products into host tissues.

Although similar mechanisms of epithelial injury have been described across intestinal and pulmonary models, their biological consequences appear to vary according to tissue context. In the intestine, MPs/NPs exposure has been associated with reduced mucus secretion, microbiota alterations, and persistent inflammatory signaling, changes that may contribute to dysbiosis and impaired mucosal homeostasis (Li et al., 2020a; Zeng et al., 2024). Experimental studies in mice and zebrafish indicate that intestinal exposure to MPs/NPs can alter microbial composition, elicit inflammatory responses, and compromise mucosal function, with evidence that these effects may depend on particle size and shape (Qiao et al., 2019).

In the skin, dermal penetration is more limited than in intestinal or pulmonary epithelia; however, nanoscale particles may access deeper cutaneous compartments, particularly through follicular routes and under conditions of barrier compromise. Experimental evidence indicates that small nanoparticles can penetrate vellus hair openings and reach epidermal antigen-presenting cells (Vogt et al., 2006), while nano-sized microplastics can be internalized by skin cells in a time- and dose-dependent manner, inducing mitochondrial oxidative stress, gasdermin D (GSDMD) recruitment to mitochondria, mtDNA release, and AIM2 inflammasome activation, thereby promoting local inflammation and impaired tissue regeneration (Wang et al., 2023; Han et al., 2024). In this context, MPs/NPs may act as carriers of chemical contaminants and other bioactive cargos, amplifying cutaneous immune perturbation and disturbing local immune homeostasis (Zhang et al., 2023b; Menichetti et al., 2024).

### 3.2 Tissue-specific co-localization of MPs/NPs and pathogens

Although the core mechanisms of epithelial injury are broadly conserved, tissue-specific microenvironments influence the probability and biological consequences of MPs/NPs–pathogen co-localization. In the gastrointestinal tract, prolonged luminal residence favors sustained contact between MPs/NPs, microbial communities, and the epithelial surface (Qiao et al., 2019), while reduced mucus secretion may further impair mucosal defense (Jin et al., 2019). In the respiratory tract, inhaled particles may enhance pathogen contact with vulnerable airway epithelia, particularly under inflammatory or remodeling-prone conditions (Lu et al., 2021; Chen et al., 2025). At the cutaneous interface, MPs/NPs may access deeper layers when barrier integrity is compromised, creating opportunities for localized interaction with chemical contaminants and other bioactive cargos. Together, these tissue-specific contexts shape how MPs/NPs interface with pathogens and host barriers across exposed epithelial surfaces (Vogt et al., 2006; Menichetti et al., 2024).

The co-localization of MPs/NPs and microorganisms is strongly influenced by particle physicochemical properties. Experimental evidence indicates that bacteria adhere preferentially to PE and PVC, compared with PP and PET, irrespective of exposure duration. This pattern suggests that characteristics such as surface hardness and related physicochemical properties govern bacterial attachment and persistence on plastic surfaces, thereby facilitating the association of MPs/NPs with pathogenic microorganisms in aquatic environments and potentially in host-associated niches (Cai et al., 2019).

This ecological interaction is reinforced by biofilm formation. PE microbeads exposed to untreated sewage become rapidly colonized by diverse bacterial assemblages that organize into dense microbial architectures on their surfaces. Metagenomic analyses further reveal progressive increases in taxonomic diversity over time, including opportunistic pathogens of veterinary and public health relevance such as *Arcobacter cryaerophilus*, *Aeromonas salmonicida*, *Vibrio areninigrae*, and *Vibrio navarrensis* (Lai et al., 2022). Thus, these findings support the concept that MPs/NPs serve as ecological platforms that favor the tissue-relevant co-localization, persistence, and transport of pathogenic microorganisms.

### 3.3 Evidence base and classification of mechanisms

To facilitate critical evaluation of the proposed framework, the evidence underpinning each mechanism has been classified according to the following categories, illustrated with representative studies: (i) direct co-exposure studies involving MPs/NPs, microorganisms, and hosts (Wu et al., 2019); (ii) studies of MPs/NPs and hosts without a microbial component (Liang et al., 2021; Qiao et al., 2021); (iii) studies of the plastisphere or microbial ecology without a host component (Sun et al., 2023); (iv) studies of cancer-associated microorganisms without plastic exposure; and (v) mechanistic analogies or hypotheses (Ying et al., 2024).

### 3.4 Enhanced permeability and microbial translocation: entry routes to immune compartments

It is important to distinguish between several mechanisms by which MPs/NPs may influence host–microbe interactions and cancer-related processes, as these pathways differ substantially in their evidence base and biological plausibility (**Table 1**). First, MPs/NPs can serve as vectors for the transport of viable environmental pathogens from contaminated ecosystems to human-exposed niches (mechanism a). Second, even in the absence of viable organisms, MPs/NPs can adsorb and concentrate microbial products such as lipopolysaccharide (LPS), which may activate host immune receptors upon particle internalization (mechanism b). Third, MPs/NPs can exert direct particle-induced epithelial injury through oxidative stress and mechanical disruption, independent of microbial cargo (mechanism c). Fourth, once epithelial integrity is compromised, translocation of endogenous microbiota (mechanism d) may occur, driving systemic inflammation and sustained immune activation. Finally, MPs/NPs often carry adsorbed chemical contaminants, including heavy metals, persistent organic pollutants, and plastic additives, that may exert independent toxic, endocrine-disrupting, or pro-carcinogenic effects (mechanism e). These mechanisms are not mutually exclusive and may operate concurrently; however, their distinct evidence bases warrant explicit consideration when interpreting the proposed framework.

Beyond co-localization, MPs/NPs may facilitate the transition from epithelial surface interaction to systemic exposure by increasing barrier permeability and facilitating microbial translocation. Upon host entry, MPs/NPs carrying plastisphere-associated biofilms may enhance microbial adhesion to epithelial surfaces and prolong host–microbe interactions. Their high surface-area-to-volume ratio and hydrophobicity favor the adsorption of opportunistic and antibiotic-resistant taxa, including *Vibrio*, *Pseudomonas*, and *E. coli*, increasing microbial survival under environmental stress and raising the likelihood of human exposure through ingestion, inhalation, or dermal contact (Zettler et al., 2013; Kirstein et al., 2016; Wu et al., 2019; Metcalf et al., 2024).

At the epithelial interface, translocation occurs through mechanisms such as tight junction disruption, endocytosis, and increased paracellular permeability. Pathogenic bacteria can exploit these vulnerabilities to penetrate the intestinal epithelium and bypass normal barrier defenses that restrict entry into systemic circulation (Shu et al., 2023). Enteric pathogens are also known to directly target tight junctions (TJ) and adherens junctions (AJ), thereby further compromising epithelial integrity and facilitating the passage of microbial cells and products across mucosal barriers (Fine et al., 2020; Shu et al., 2023).

In the intestine, combined exposure to polystyrene MPs/NPs exacerbates barrier disruption through ROS-mediated epithelial cell death, facilitating particle passage across the mucosa (Liang et al., 2021). In airway epithelial models, PS MPs/NPs reduce barrier integrity by inducing oxidative imbalance and depleting TJ proteins (Yang et al., 2021); co-exposure with inhaled allergens further enhances airway permeability (Hu et al., 2025). Photoaged PVC microplastics aggravate airway barrier dysfunction via TXNIP–NLRP3-mediated inflammatory amplification (Liu et al., 2026b). As barrier function deteriorates, plastic particles and microbial products may gain access to immune compartments and systemic circulation, potentially prolonging host exposure to microbial-derived stimuli (Wang et al., 2026).

Barrier failure and microbial translocation may extend the consequences of epithelial injury beyond local tissues by permitting microbial cells and products to access extraintestinal and immune compartments. Evidence from barrier biology indicates that translocation of commensal and pathobiont bacteria across impaired mucosal surfaces can sustain systemic immune activation and contribute to chronic inflammatory disease states, even in the absence of overt infection (Fine et al., 2020). Within the proposed framework, MPs/NPs could function not only as epithelial disruptors but also as ecological facilitators of microbial persistence and delivery, since their surfaces can adsorb pathogens and support biofilm formation, potentially increasing the likelihood of prolonged host exposure to translocatable microbial cargos (Sinha et al., 2025). Together, these observations support the hypothesis that MPs/NPs-associated barrier dysfunction and microbial translocation could contribute to chronic inflammatory signaling and immune dysregulation, with potential implications for conditions relevant to tumor biology.

## 4 Immune dysregulation and tumor microenvironment reprogramming

Exposure to MPs/NPs has been associated with immune dysfunction in experimental models, with evidence indicating effects on macrophage and lymphocyte function and the promotion of chronic low-grade inflammation (Alijagic et al., 2023; Fan and Ha, 2025). A frequently proposed mechanism involves activation of inflammasome-dependent pathways, particularly NLRP3, which can drive release of pro-inflammatory cytokines such as IL-1β and may contribute to an inflammatory milieu (Kelley et al., 2019; Alijagic et al., 2023).

Beyond systemic immune alterations, MPs/NPs have been linked to structural and tissue remodeling in experimental settings. Emerging evidence suggests that these particles may influence the composition and organization of the extracellular matrix (ECM) and stromal compartments, with potential implications for cell migration and tissue permissiveness (Huang et al., 2023; Kim et al., 2026). In addition, MPs/NPs may adsorb and transport persistent organic pollutants and heavy metals, which could act as endocrine disruptors and potentially influence carcinogenesis-related processes (Sun et al., 2023). In summary, these observations support the hypothesis that MPs/NPs could contribute to chronic inflammation, immune dysregulation, and tissue remodeling, with potential relevance to tumor biology.

### 4.1 MPs/NPs as amplifiers of host–microbe signaling

MPs/NPs can function as biologically active interfaces that concentrate microbial ligands while simultaneously inducing intrinsic cellular stress responses. Through the adsorption of pathogen-associated molecular patterns (PAMPs), such as LPS, onto their surfaces, MPs/NPs increase the local density and persistence of immunostimulatory signals, thereby enhancing sustained interactions with host immune cells (Yang et al., 2023a). This effect is further reinforced within the plastisphere, where biofilm-associated microbial communities function as continuous reservoirs of immune-activating ligands.

At the host interface, PRRs are central to the detection of microbial and damage-related signals. Membrane and endosomal Toll-like receptors (TLRs) recognize conserved extracellular or endocytosed pathogen-associated motifs, whereas cytosolic sensors such as nucleotide-binding oligomerization domain-like receptors (NLRs) and RIG-I-like receptors (RLRs) detect intracellular pathogen-associated molecular patterns and danger-associated signals. Together, these receptor systems initiate signaling cascades that activate NF-κB and interferon regulatory factors (IRFs), thereby inducing pro-inflammatory cytokine production, immune cell recruitment, and, in the case of inflammasome-forming NLRs, the maturation of IL-1β and IL-18 (Kawai and Akira, 2010; Takeuchi and Akira, 2010; Latz et al., 2013).

Among these pathways, the NLRP3 inflammasome emerges as a central integrative node linking microbial inputs and MPs/NPs-induced cellular stress. TLR-mediated NF-κB activation provides the priming signal through upregulation of NLRP3 and pro-IL-1β, whereas MPs/NPs generate multiple secondary activation signals that converge on inflammasome assembly. Oxidative stress is a major trigger, as MPs/NPs induce mitochondrial ROS production, disrupt electron transport chain function, and deplete antioxidant defenses, thereby promoting caspase-1 activation and IL-1β maturation (Busch et al., 2022; Chen et al., 2024). In line with this, PS-NPs have been shown to induce hepatotoxicity through the NRF2-NLRP3 signaling pathway in mice (Wen et al., 2024). Experimental evidence further shows that amino-modified polystyrene MPs/NPs (~100 nm) induce ROS-dependent NLRP3 activation in primary human macrophages, an effect that is abrogated by ROS scavenging (Lunov et al., 2011). Mitochondrial dysfunction amplifies this response by promoting loss of membrane potential and organelle fragmentation, which can release mitochondrial DNA (mtDNA) as a potent DAMP capable of directly activating NLRP3 (Li et al., 2023; Wang et al., 2023).

In addition, lysosomal destabilization following particle internalization leads to cathepsin release into the cytosol, constituting an additional activation pathway analogous to classical particulate toxicants such as silica and asbestos (Lunov et al., 2011; Zhan et al., 2023). Importantly, these physicochemical signals are synergistically reinforced by plastisphere-associated microbial ligands, as MPs/NPs-bound LPS enhances TLR4/NF-κB signaling and increases cellular sensitivity to secondary inflammasome activation signals (He et al., 2022; Yang et al., 2023a). Beyond inflammasome activation, MPs/NPs also induce alternative lytic cell death pathways, particularly necroptosis. Under conditions of caspase-8 inhibition, receptor-interacting protein kinases RIPK1 and RIPK3 activate mixed lineage kinase domain-like protein (MLKL), leading to membrane rupture and release of intracellular DAMPs (Dhuriya and Sharma, 2018; Wu et al., 2025a). Notably, caspase-8 deficiency has been shown to facilitate NLRP3 inflammasome assembly through RIPK1/RIPK3/MLKL signaling (Kang et al., 2013). These events reinforce inflammatory signaling through feed-forward amplification loops and further sustain PRR activation (Ye et al., 2024).

As illustrated in **Figure 4**, MPs/NPs could function as integrative immunological amplifiers that couple microbial ligand presentation with intracellular stress signaling. This dual mechanism potentially sustains PRR activation, chronic inflammasome signaling, and persistent inflammatory responses, thereby promoting epithelial dysfunction, microbial translocation, immune dysregulation, and pro-tumorigenic conditions.

**Figure 4:**
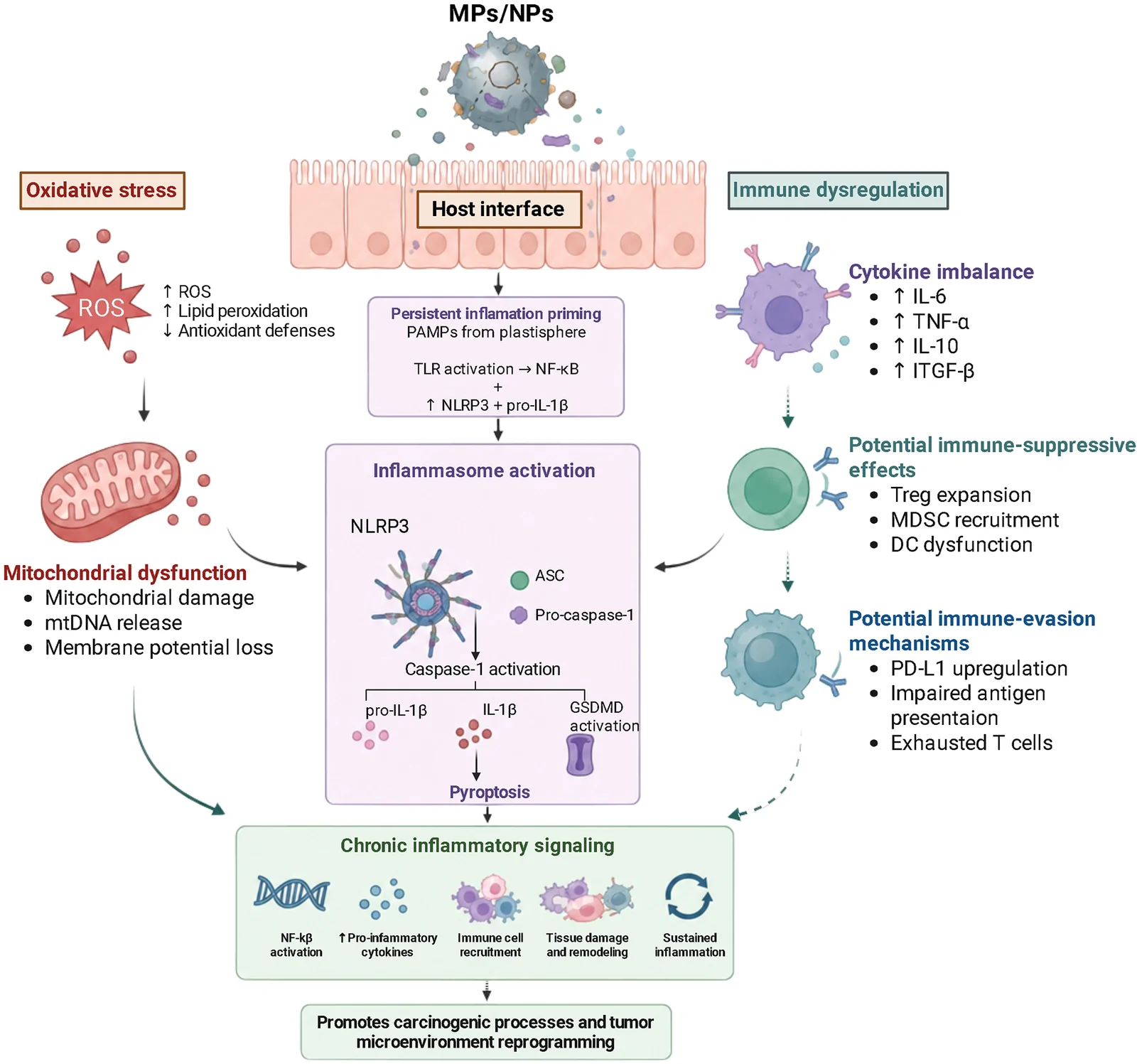
Proposed molecular pathways linking MPs/NPs exposure with inflammasome activation, immune dysregulation, and chronic inflammatory signaling. At the host interface, plastisphere-associated PAMPs may provide inflammatory priming through TLR-mediated NF-κB activation, with subsequent increases in NLRP3 and pro-IL-1β. In parallel, MPs/NPs exposure may be associated with oxidative stress, lipid peroxidation, mitochondrial injury, mitochondrial DNA release, and loss of membrane potential, which may provide secondary signals for inflammasome activation. These pathways converge on NLRP3 inflammasome assembly, caspase-1 activation, IL-1β maturation, gasdermin D activation, and pyroptotic cell death in relevant experimental contexts. Immune dysregulation may involve altered cytokine profiles, regulatory T-cell expansion, myeloid-derived suppressor cell recruitment, and dendritic-cell dysfunction. Potential immune-evasion mechanisms include PD-L1 upregulation, impaired antigen presentation, and T-cell dysfunction/exhaustion. Solid arrows indicate mechanisms supported by direct experimental evidence in the reviewed literature, whereas dashed arrows indicate proposed or inferred mechanisms supported primarily by indirect or context-dependent evidence. These interconnected processes may contribute to persistent inflammatory signaling, immune-cell recruitment, tissue damage and remodeling, and, potentially, tumor microenvironment reprogramming and carcinogenic processes. (Created with BioRender.com).

### 4.2 Chronic inflammation and immune evasion

Chronic MP/NP exposure has been associated with sustained inflammatory responses in experimental models, including oxidative stress, epithelial injury, and activation of innate immune pathways. Microbial-derived stimuli provide an additional source of inflammatory signaling through PRRs and inflammasome pathways. These pathways contribute to the production of mediators such as IL-6 and TNF-α, which can maintain a chronic inflammatory environment capable of disrupting immune homeostasis (Kelley et al., 2019; Chen et al., 2025).

Sustained inflammation is closely linked to impaired antitumor immune surveillance and the development of immunosuppressive tumor microenvironments. Chronic inflammatory signaling favors macrophage phenotypes with immunosuppressive, pro-angiogenic, and tissue-remodeling properties and can support conditions in which transformed cells more readily escape immune clearance. Studies using tumor-derived microparticles further illustrate how particulate signaling can simultaneously induce IL-1β release through TLR3/NLRP3 activation and promote inflammatory and immunosuppressive macrophage states, although these observations arise from a biologically distinct particulate system and should not be directly extrapolated to environmental MPs/NPs (Chen et al., 2020). These alterations create a permissive environment in which transformed cells are more likely to evade clearance and persist.

A plausible mechanistic bridge between MP/NP-associated inflammation and tumor immune regulation is the IL-6/JAK/STAT3 axis. Persistent STAT3 signaling is well established as a regulator of tumor cell proliferation, survival, angiogenesis, and immune suppression, while also promoting the accumulation and activity of immunosuppressive myeloid and stromal populations within the tumor microenvironment (Wang et al., 2018; Zou et al., 2020; Hashimoto et al., 2022; Thuya et al., 2025). Thus, rather than demonstrating a direct MP/NP-driven pathway to immune evasion, current evidence supports a model in which inflammatory signals associated with particle exposure could intersect with established tumor-promoting signaling networks such as STAT3.

An additional immune-modulating layer is introduced by the rapid formation of a biomolecular corona when MPs/NPs encounter biological fluids. This corona, enriched with immunoglobulins, complement components, and acute-phase proteins, alters immune recognition in a context-dependent manner: some components enhance cellular uptake through opsonization, whereas others may mask antigenic surfaces, prolong circulation time, and facilitate immune evasion (Caracciolo et al., 2015; Visalakshan et al., 2019; Cao et al., 2022; Fu et al., 2025; Le et al., 2025). Critically, the formation of this corona may displace, occlude, or otherwise modulate the presentation of microbial ligands originally adsorbed onto MPs/NPs surfaces from environmental sources, suggesting that the biological interface presented to host immune cells may diverge substantially from the original plastisphere community. Thus, these mechanisms may establish a feed-forward loop in which chronic inflammation and immune escape reinforce one another, potentially positioning MPs/NPs as mediators linking xenobiotic exposure with systemic immune dysfunction and malignant progression (**Table 3**).

**Table 3:** Mechanisms of immune dysregulation induced by MPs/NPs.

Component/process	Mechanism of action of MPs/NPs	Immunological consequence	References
PAMPs/DAMPs Amplification	Adsorption of microbial ligands such as LPS and formation of the plastisphere.	Increased local density of immunostimulatory signals and sustained activation of pattern recognition receptors such as NF-κB/NLRP3 pathway and NRF2-NLRP3.	(Cao et al., 2023b; Wen et al., 2024)
TLR/NF-κB Axis	Activation of surface TLR4 and endosomal receptors.	Inflammatory priming and transcription of pro-inflammatory cytokines (TNF-α, IFN-γ, IL-2, IL-6, IL-8, IL-1β).	(Cao et al., 2023b; Zhang et al., 2023a; Xu et al., 2024).
NLRP3 Inflammasome	Induction of oxidative stress and secondary signals following particle internalization.	IL-1β maturation via caspase-1 activation and pyroptotic cell death (canonical pathway by gasdermin D-mediated).	(Alijagic et al., 2023)
Cell Death Pathways	Cellular damage by activation of RIPK1/RIPK3 and MLKL under caspase-8 inhibition.	Necroptosis, leading to DAMP release and amplification of pro-inflammatory feedback loops.	(Kang et al., 2013; Ye et al., 2024)

### 4.3 Reprogramming of the tumor microenvironment

MPs/NPs have been associated with tissue remodeling in experimental models through interconnected immunological, stromal, and physicochemical mechanisms. Acting as reactive biomolecular interfaces that can adsorb microbial ligands and anthropogenic toxicants, these particles may sustain chronic inflammatory signaling, which could in turn influence tissue structure and cellular behavior (Huang et al., 2023; Kim et al., 2026). These changes, observed primarily in preclinical settings, include alterations in extracellular matrix organization and biomechanical properties that have been linked to enhanced cell migration in cancer models (Kim et al., 2026). These observations correspond to mechanism (c) in **Table 1**, direct particle-induced epithelial injury and subsequent tissue remodeling.

In parallel, MPs/NPs may influence immune cell phenotypes within tissues. Sustained inflammatory signaling and exposure to particle-associated immunogenic stimuli could favor macrophage polarization toward phenotypes associated with immunosuppressive and tissue-remodeling functions. Supporting this broader concept, particle-mediated signaling has been shown to induce macrophage states in which pro-inflammatory outputs coexist with immunosuppressive functions, a pattern observed in tumor microenvironments (Chen et al., 2020; Hu et al., 2021; Fan and Ha, 2025). In addition to these microbial and particle-driven mechanisms, MPs/NPs may exert effects through adsorbed chemical contaminants (mechanism e), which can independently influence tissue homeostasis.

At the molecular level, STAT3 has been proposed as a potential node linking chronic inflammation to tissue remodeling and oncogenic processes. Persistent STAT3 activation, driven by inflammatory mediators such as IL-6, can promote proliferation, survival, angiogenesis, and immunotolerance (Piao et al., 2016; Zou et al., 2020; Hashimoto et al., 2022). Whether MPs/NPs directly activate STAT3 *in vivo* remains to be established, but this pathway represents a biologically plausible mechanism by which particle-induced inflammation could influence cancer-relevant processes (Hu et al., 2024).

MPs/NPs may also act as vectors for persistent organic pollutants and heavy metals, which have endocrine-disrupting and potentially carcinogenic properties. Following systemic internalization, these co-transported xenobiotics could intensify oxidative stress and genomic instability, potentially compounding tissue remodeling and inflammation (Zhang and Xu, 2022; Sun et al., 2023). Together, these observations support the hypothesis that MPs/NPs could influence TME-related processes by integrating chronic inflammation, stromal reorganization, and immune modulation (**Figure 5**).

**Figure 5:**
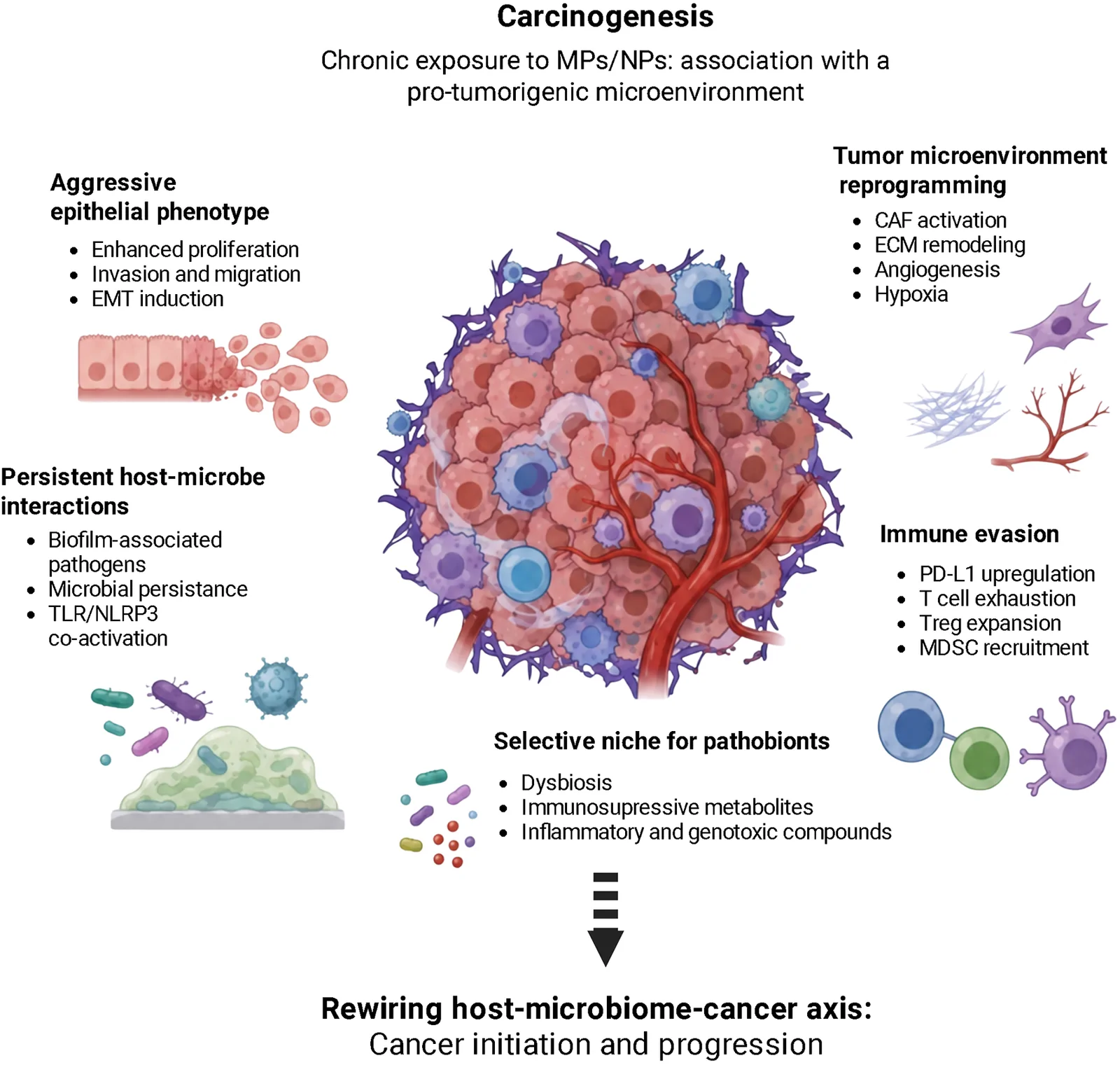
Oncogenic outcomes of MPs/NPs exposure: tumor microenvironment reprogramming and host–microbiome–cancer axis disruption. Persistent host–pathogen interactions, including biofilm-associated pathogens, microbial persistence, and TLR/NLRP3 co-activation, contribute to the establishment of a selective niche enriched in dysbiosis, immunosuppressive metabolites, and inflammatory or genotoxic compounds. These signals converge with aggressive epithelial changes, including enhanced proliferation, invasion, migration, and epithelial–mesenchymal transition (EMT), while simultaneously promoting TME remodeling through cancer-associated fibroblast activation, ECM remodeling, angiogenesis, and hypoxia. In parallel, immune evasion mechanisms such as PD-L1 upregulation, T-cell exhaustion, regulatory T-cell expansion, and myeloid-derived suppressor cell (MDSC) recruitment reinforce tumor-permissive conditions. These interconnected processes rewire the host–microbiome–cancer axis and contribute to tumor initiation, progression, therapy resistance, metastasis, and poor prognosis. (Created with BioRender.com).

## 5 Dysbiosis, metabolic rewiring, and oncogenic pathways

Exposure of the gastrointestinal tract to MPs/NPs disrupts host microbiome homeostasis through a variety of interconnected mechanisms, including direct interference with the microbiota, impairment of intestinal barrier function, activation of the immune system, and altered production of microbial metabolites (Hernández et al., 2025). In 2019, Schwabl et al. first reported the presence of MPs/NPs in human feces (Schwabl et al., 2019), suggesting their accumulation within the digestive system and highlighting a potentially important threat to human health. Consequently, the ingestion of MPs/NPs via contaminated water and dietary matrices represents a burgeoning concern within the scientific community, especially considering that this constitutes the main route of entry into the body and that these particles may accumulate in the digestive tract (Osman et al., 2023). Among the main consequences described are mucosal damage, increased permeability, inflammation, and profound gut microbiota dysbiosis (Qiao et al., 2019).

### 5.1 Microbiome dysbiosis and microbe-mediated carcinogenesis in the context of MPs/NPs

The intestinal microbiome is a highly dynamic ecosystem composed of trillions of microorganisms that closely interact with host physiology, metabolism, and immune function (Lloyd-Price et al., 2016). Under homeostatic conditions, microbial communities are maintained through cooperative and competitive interactions that preserve barrier integrity, metabolic balance, and immune regulation. Key taxa such as *Faecalibacterium prausnitzii* and *Akkermansia muciniphila* contribute to epithelial function and immunological tolerance through the production of short-chain fatty acids (SCFAs), particularly butyrate, which serves as a major energy source for colonocytes and modulates inflammatory responses (Flint et al., 2015; Sutton and Hills, 2025).

Disruption of this ecological equilibrium, commonly referred to as dysbiosis, has been strongly associated with carcinogenesis. Alterations in microbial composition and diversity can reshape the tumor microenvironment, sustain chronic inflammation, and increase carcinogenic metabolites that contribute to tumor initiation and progression (Wong and Yu, 2019; Duan et al., 2024). Reduced SCFA production, for example, is associated with elevated levels of harmful metabolites such as secondary bile acids, which induce DNA damage, oxidative stress, and activation of oncogenic pathways including NF-κB and WNT, thereby promoting cellular proliferation and survival (Morrison and Preston, 2016; Yang and Qian, 2022; Du et al., 2024).

In addition to metabolic disruption, specific microbial taxa contribute directly to tumorigenesis through pathogenic and immunomodulatory mechanisms. Associations have been established between *Helicobacter pylori* and gastric cancer, and *Fusobacterium nucleatum* and colorectal cancer (Ou et al., 2023; Salvatori et al., 2023; Zhang et al., 2025b). More broadly, microbial oncogenesis involves contact-dependent interactions, microbe-derived metabolites, and immune-mediated mechanisms that collectively alter host signaling, impair immunosurveillance, and favor neoplastic transformation (Garrett, 2015; White and Wargo, 2020; Sevcikova et al., 2023). Certain microorganisms may also influence tumor metabolism and therapeutic resistance; for instance, *Lactobacillus iners* has been associated with aggressive tumor phenotypes through increased lactate production and reduced sensitivity to chemoradiotherapy (Colbert et al., 2023).

Within the context of MPs/NPs exposure, these processes acquire an additional ecological dimension. MPs/NPs can act as microbiome modifiers by promoting biofilm formation, altering taxonomic composition, and enriching pathobionts and antibiotic-resistant taxa. By serving as vectors for microbial communities and their metabolites, these particles may exacerbate dysbiosis and sustain oncogenic host–microbe interactions. Thus, the microbiome may emerge as a pivotal mediator linking MPs/NPs exposure to chronic inflammation, metabolic rewiring, and carcinogenic signaling pathways.

### 5.2 Disruption of the gut microbiome by MPs/NPs exposure

Experimental studies indicate that gastrointestinal exposure to MPs/NPs can alter gut microbial composition, although the magnitude and direction of these changes vary across particle types and biological models (Bora et al., 2024). In mice, repeated oral exposure to polystyrene MPs/NPs induced marked microbial dysbiosis together with intestinal barrier injury, including enrichment of opportunistic taxa and depletion of bacteria associated with barrier-supportive functions (Jin et al., 2018; Qiao et al., 2021). In zebrafish, exposure to polystyrene similarly induced changes in microbial richness and composition accompanied by intestinal inflammatory responses (Jin et al., 2018). These findings provide experimental support for an effect of MPs/NPs exposure on gut microbial ecology, while also highlighting substantial model- and particle-dependent variability.

The functional consequences of these shifts are also heterogeneous. MPs/NPs can directly affect bacterial growth and metabolism under controlled conditions, as shown by dose-dependent growth inhibition and broad metabolic alterations in representative gut bacterial strains exposed to polystyrene particles (Chi et al., 2025). In more complex human gut simulation systems, repeated PE MPs/NPs exposure altered microbial composition and metabolic activity, including enrichment of potentially harmful pathobionts and, in infant models, reduced butyrate production (Fournier et al., 2022, 2023). Importantly, however, these microbial changes were not accompanied by significant microbiota-mediated impairment of intestinal barrier integrity in either model, indicating that dysbiosis does not necessarily translate into measurable epithelial dysfunction. Polymer-specific effects are also evident, as biodegradable PCL and PLA MPs undergo partial gastrointestinal transformation and induce distinct changes in microbial diversity and probiotic taxa (Peng et al., 2024).

Interpretation of specific taxonomic signatures requires particular caution. Although Firmicutes and Bacteroidetes contribute substantially to carbohydrate fermentation and SCFA production, their relative abundance varies considerably across physiological and pathological states, and the Firmicutes/Bacteroidetes ratio is not considered a consistent marker of dysbiosis or disease (Andoh, 2016; Magne et al., 2020). Similar caution applies when relating MPs/NPs-associated microbial changes to taxa previously associated with inflammatory disorders such as IBS or Crohn’s disease, since these clinical associations do not establish equivalent pathological effects following MPs/NPs exposure (Assa et al., 2016; Zhou and Zhi, 2016; Pittayanon et al., 2019; Parker et al., 2020). Human evidence remains comparatively limited: observational studies have identified associations between MPs/NPs burden and specific microbial taxa in stool, placenta, and meconium, but have not demonstrated a consistent global microbiome signature or a causal relationship between MPs/NPs exposure and human dysbiosis (Nugrahapraja et al., 2022; Liu et al., 2023; Yang et al., 2026). Collectively, current evidence supports an effect of MPs/NPs on microbial composition and function in experimental systems, while the biological significance, reproducibility, and translational relevance of these alterations remain strongly dependent on particle characteristics, host context, and experimental design.

### 5.3 MPs/NPs-driven microbiome dysbiosis and metabolic reprogramming

MPs/NPs have been proposed as ecological disruptors of the intestinal microbiome, potentially altering the physicochemical and nutritional conditions that sustain microbial homeostasis (Fackelmann and Sommer, 2019). Their accumulation within the intestinal lumen, together with their capacity to adsorb xenobiotics, may reshape microbial growth dynamics and favor the expansion of stress-adapted and pro-inflammatory taxa (Fackelmann and Sommer, 2019; Li et al., 2025b). These effects may be driven by oxidative and inflammatory conditions that selectively favor nitrate-respiring *Enterobacteriaceae* and other *Proteobacteria*, thereby sustaining chronic low-grade inflammation and establishing niches that permit pathogen persistence, as reflected by the increased abundance of *Desulfovibrionaceae*. This shift may also involve a decline in beneficial taxa such as Clostridia_UCG-014, *Roseburia*, and *Akkermansia*, alongside an expansion of the pathogenic Desulfovibrionaceae following PS-NP exposure (Ni et al., 2017; Yang et al., 2025).

Beyond compositional changes, MPs/NPs have been associated with alterations in microbial metabolism. Untargeted metabolomics has revealed changes in intracellular metabolites and the exometabolome, suggesting broad disruption of microbial metabolic networks (Zhang et al., 2024; Chi et al., 2025). Metagenomic and metatranscriptomic analyses have revealed disruptions in metabolic pathways, including those involved in nitrogen transformation and oxidative stress responses, suggesting broad metabolic dysregulation within exposed microbial consortia (Yang et al., 2022; Zhang et al., 2024).

Thereby, these observations support the hypothesis that MPs/NPs may influence microbiome dysbiosis not only through ecological selection but also through metabolic alterations. By affecting microbial viability, metabolite production, and adaptive stress responses, MPs/NPs could create a pro-inflammatory and metabolically altered environment that favors microbial persistence and sustained inflammatory signaling, with potential relevance to conditions associated with chronic disease risk.

### 5.4 Molecular pathways underlying MPs/NPs-driven inflammation and tumor progression

MPs/NPs exposure has been associated with the activation of interconnected molecular pathways that link oxidative stress and cellular injury to fibrosis and inflammatory persistence. Among these, the cGAS–STING axis has been proposed as a route through which intracellular damage may be translated into sustained inflammatory signaling. Internalization of MPs/NPs can disrupt redox homeostasis and mitochondrial function, potentially leading to genomic damage and cytosolic release of double-stranded DNA. This may activate cyclic GMP–AMP synthase (cGAS), promote cGAMP production, and trigger STING-dependent signaling, ultimately increasing IRF3 and NF-κB activity and reinforcing inflammatory responses (Dunphy et al., 2018; Decout et al., 2021; Shen et al., 2022).

In parallel, MPs/NPs-induced oxidative stress has been linked to activation of the Wnt/β-catenin pathway, a regulator of tissue repair and fibroblast activation. Persistent stimulation of this axis has been shown to promote the expression of pro-fibrotic mediators such as TGF-β and fibronectin in experimental models, suggesting a connection between chronic tissue injury and stromal remodeling (Song et al., 2015; Li et al., 2020b, 2022; An et al., 2021). This mechanism may be relevant to conditions involving fibrotic and inflammatory tissue changes.

MPs/NPs have also been associated with endoplasmic reticulum stress, potentially activating the GRP78/CHOP/Bcl-2 signaling axis (Zhao et al., 2025). Under stress conditions, dissociation of GRP78 from ER sensor proteins may initiate downstream pathways that promote CHOP activation, disrupt the Bcl-2/Bax balance, and drive mitochondrial dysfunction and apoptosis. Together, these observations suggest that MPs/NPs may engage coordinated molecular programs that integrate stress signaling, inflammatory responses, and fibrotic remodeling. Through cGAS–STING, Wnt/β-catenin, and GRP78/CHOP/Bcl-2 signaling, MPs/NPs could contribute to persistent pathological states characterized by chronic inflammation and tissue remodeling, with potential implications for conditions relevant to tumor biology (Ding et al., 2024) (**Table 4**).

**Table 4:** MPs/NPs-driven tumor microenvironment reprogramming and oncogenic pathways.

Reprogramming/oncogenic dimension	MPs/NPs-related mechanism	Microbial/molecular mediator	Tumor-relevant consequence	References
Macrophage Polarization	MPs/NPs-associated inflammatory signaling promotes phenotypic skewing toward M2-like TAMs.	*Fusobacterium nucleatum*, *Escherichia coli pks+*, *Porphyromonas gingivalis*	Immunosuppression, tissue remodeling, angiogenic support, and tumor growth promotion.	(Hu et al., 2021; Lee et al., 2025)
Extracellular matrix remodeling	MPs/NPs disrupt the structural and mechanical organization of the extracellular matrix and favor stromal permissiveness.	*Fusobacterium nucleatum*, *Bacteroides fragilis*, *Helicobacter pylori*	Enhanced invasion, migration, and establishment of a mechanically permissive tumor stroma.	(Huang et al., 2023)
Oncogenic inflammatory signaling	Persistent inflammatory cues induced by MPs/NPs converge on IL-6/STAT3, NF-κB, and related pro-survival pathways.	*Fusobacterium nucleatum*, *Helicobacter pylori*.	Increased proliferation, anti-apoptotic survival, angiogenesis, and genomic instability.	(Piao et al., 2016; Chen et al., 2024)
Biocorona-mediated immune modulation	Adsorption of host-derived proteins, including immunoglobulins, complement factors, and albumin, reshapes host–particle interactions.	*Pseudomonas aeruginosa*, *Staphylococcus aureus*, context-dependent protein-coated surface interactions.	Altered cellular uptake, modified immune recognition, and immune evasion-like phenotypes.	(Caracciolo et al., 2015; Visalakshan et al., 2019; Le et al., 2025)
Trojan Horse Effect	MPs/NPs co-transport POPs and heavy metals together with microbial signals.	*H. pylori, Staphylococcus* aureus	Endocrine disruption, oxidative stress, carcinogenic promotion, and metastasis support.	(Zhang and Xu, 2022; Sevcikova et al., 2023; Duan et al., 2024)
SCFA depletion and metabolic rewiring	Dysbiosis of *Firmicutes* and *Bacteroidetes* reduces SCFA production and disrupts host metabolic homeostasis.	SCFAs; secondary bile acids	Loss of anti-inflammatory signaling, impaired colonocyte energy supply, and enrichment of genotoxic metabolites.	(Du et al., 2024)
Gastric mucosal barrier disruption	MPs/NPs, particularly PE particles, facilitate biofilm-associated interaction with *H. pylori* and damage epithelial tight junctions.	*Helicobacter pylori*; tight junction proteins	Deepened tissue invasion, amplified gastric inflammation, epithelial erosion, and goblet cell depletion.	(Zhang et al., 2025b)
Genotoxic signaling	MPs/NPs activate oncogenic pathways also exploited by microbial carcinogens.	Wnt/β-catenin	Uncontrolled proliferation, fibrosis, and survival of transformed cells.	(Song et al., 2015; Li et al., 2022; Ding et al., 2024)
Stress-response signaling	MPs/NPs-induced mitochondrial injury promotes cytosolic DNA sensing and chronic inflammatory amplification.	cGAS–STING axis	Feed-forward inflammation, fibrotic progression, and tumor-supportive signaling.	(Dunphy et al., 2018; Shen et al., 2022)
Inflammasome and inflammatory pathway convergence	MPs/NPs and microbial stimuli reinforce persistent inflammatory signaling.	NF-κB, NLRP3, STAT3; *Helicobacter pylori*.	Sustained pro-tumorigenic inflammation, enterohepatic injury, and oncogenic microenvironmental conditioning.	(Chen et al., 2024; Xu et al., 2024)

## 6 Translational perspectives and research horizons

### 6.1 From experimental models to ecological carcinogenesis

Traditional monoculture systems have provided foundational insights into the cytotoxic and inflammatory cascades triggered by MPs/NPs; however, they may not fully capture the ecological complexity of host–microbiota–environment interactions relevant to cancer-related processes. Emerging multi-component platforms, particularly triple co-culture systems incorporating xenobiotics, microbial consortia, and host epithelial or immune lineages, together with organoid architectures and microfluidic “gut-on-a-chip” technologies, may help bridge this gap by recreating physiologically relevant microenvironments. These systems enable the simultaneous analysis of microbial colonization, biofilm dynamics, epithelial barrier kinetics, microbial translocation, and key immune signaling pathways under controlled conditions (Kim, 2023; Penarete-Acosta et al., 2024; De Martinis et al., 2025). Importantly, they allow MPs/NPs to be modeled as potential ecological mediators linking microbial communities, epithelial responses, and immune signaling.

In various experimental models, MPs/NPs have been associated with cellular changes relevant to cancer biology. In breast cancer cell lines such as MDA-MB-231 and MCF-7, exposure to polypropylene-derived particles has been shown to alter the expression of cell cycle–related genes, including *TMBIM6*, *AP2M1*, and *PTP4A2*, while downregulating *FTH1*, changes accompanied by increased IL-6 secretion and pro-inflammatory signaling (Park et al., 2023). Similarly, PS MPs/NPs exhibit size- and cell type–dependent internalization, with some experimental settings showing stimulatory effects on proliferation and migration (Schnee et al., 2024).

In glioblastoma models, polyethylene MPs/NPs have been associated with increased proliferation, migration, and spheroid formation, suggesting a potential role for chronic exposure in cellular changes relevant to tumor progression (Rafazi et al., 2024). In cutaneous squamous cell carcinoma cell lines, MPs/NPs have been shown to induce mitochondrial ROS generation, disrupt membrane potential, and activate the NLRP3 inflammasome, effects associated with tumor cell proliferation *in vitro* while also causing damage to normal skin cells (Wang et al., 2023). In colorectal cancer, tissue-specific accumulation of MPs/NPs has been associated with increased tumor incidence in animal models and acquired chemoresistance through mTOR/ULK1-mediated protective autophagy and microbiota-driven inflammation (Pan et al., 2025).

Together, these findings support a conceptual shift from classical substance-centered toxicology toward an ecological framework in which MPs/NPs may act as integrative interfaces linking environmental exposure, microbial dynamics, and cancer-related cellular processes. However, direct evidence in human populations remains limited, and the proposed framework should be considered hypothesis-generating.

### 6.2 Biomarker frameworks for MP-driven disease and cancer risk

The identification of measurable biological responses is important for translating mechanistic insights into clinically and epidemiologically relevant research on MPs/NPs exposure. However, the markers discussed in this section should not be interpreted as validated biomarkers of MP/NP exposure or cancer risk. Most represent nonspecific indicators of inflammation, oxidative stress, epithelial injury, or barrier dysfunction. Their potential translational value therefore lies primarily in their use as candidate mechanistic readouts for characterizing biological responses associated with MPs/NPs exposure and for informing future biomarker development.

Inflammatory mediators, particularly the inflammasome-associated cytokines IL-1β and IL-18, may provide useful readouts of innate immune activation in experimental models exposed to MPs/NPs (Kelley et al., 2019; Alijagic et al., 2023). Similarly, oxidative stress-related measures, including malondialdehyde and 4-hydroxynonenal, together with changes in antioxidant defenses such as superoxide dismutase and glutathione peroxidase, may reflect altered redox homeostasis following exposure (Viana et al., 2025). These indicators can help characterize inflammatory and oxidative responses potentially associated with MPs/NPs, but their lack of specificity precludes their interpretation as exposure-specific biomarkers or independent predictors of cancer risk.

A critical limitation in the field is the absence of validated biomarkers specifically indicative of MP/NP exposure. Currently, no biomarker has been demonstrated to reflect MP/NP exposure with sufficient specificity or reproducibility for clinical or epidemiological use. Experimental models of chronic PS-NP exposure have demonstrated intestinal mechanical and immune barrier dysfunction (Li et al., 2024a), and markers related to epithelial integrity, such as zonulin, lipopolysaccharide-binding protein, and intestinal fatty acid-binding protein, have been proposed as nonspecific readouts of barrier dysfunction rather than as biomarkers of MP/NP exposure per se. These markers may provide complementary mechanistic information, but their lack of specificity for MP/NP exposure limits their interpretation as exposure-specific biomarkers or independent predictors of cancer risk. Their potential use for early detection, risk stratification, or monitoring of MPs/NPs-associated disease remains hypothetical and requires validation in well-characterized human populations, including assessment of specificity, reproducibility, exposure–response relationships, and clinical relevance.

### 6.3 MPs/NPs, environmental resistome and tumor microenvironment

A critical translational dimension of MPs/NPs exposure is the emergence of the environmental resistome within plastisphere-associated biofilms. MPs/NPs can create dense ecological niches in which antimicrobial resistance genes (ARGs), virulence factor genes, opportunistic pathogens, and co-selective pollutants converge, potentially increasing the likelihood of exposure to high-risk microbial configurations. Experimental evidence indicates that MPs/NPs can enrich microbial communities carrying ARGs and virulence-associated genes, with positive correlations observed between dysbiosis and the dissemination of harmful genetic determinants (Xia et al., 2025). Plastisphere-associated communities exhibit distinctive resistomes and can harbor opportunistic pathogens not detected on natural substrates, suggesting that MPs/NPs may function as selective interfaces that reorganize microbial ecology toward persistence and pathogenicity (Wu et al., 2019). This process may be further reinforced by habitat-specific ARG dissemination and mobile genetic element–mediated transfer, which could enhance the role of the plastisphere as a reservoir of resistance and virulence traits (Bao et al., 2025).

This ecological convergence may have relevance for host biology. Persistent colonization by resistant pathobionts has been associated with low-grade inflammation and impaired microbial clearance in experimental models, potentially prolonging microbial persistence within host-associated niches (Fine et al., 2020; Li et al., 2025b). The co-localization of ARGs with virulence determinants within plastisphere biofilms may also increase pathogenic potential, as microbial communities enriched in toxins, adhesion factors, and biofilm regulators could support immune evasion and chronic inflammatory persistence.

At the tissue level, MPs/NPs exposure has been associated with activation of innate immune pathways such as NF-κB and NLRP3 (He et al., 2022; Xu et al., 2024). In parallel, PS-MPs may induce inflammatory responses and promote M2-associated cytokine expression in lung tissues, suggesting that chronic exposure may contribute to tissue remodeling and fibrosis (Lee et al., 2025) (**Figure 6**).

**Figure 6:**
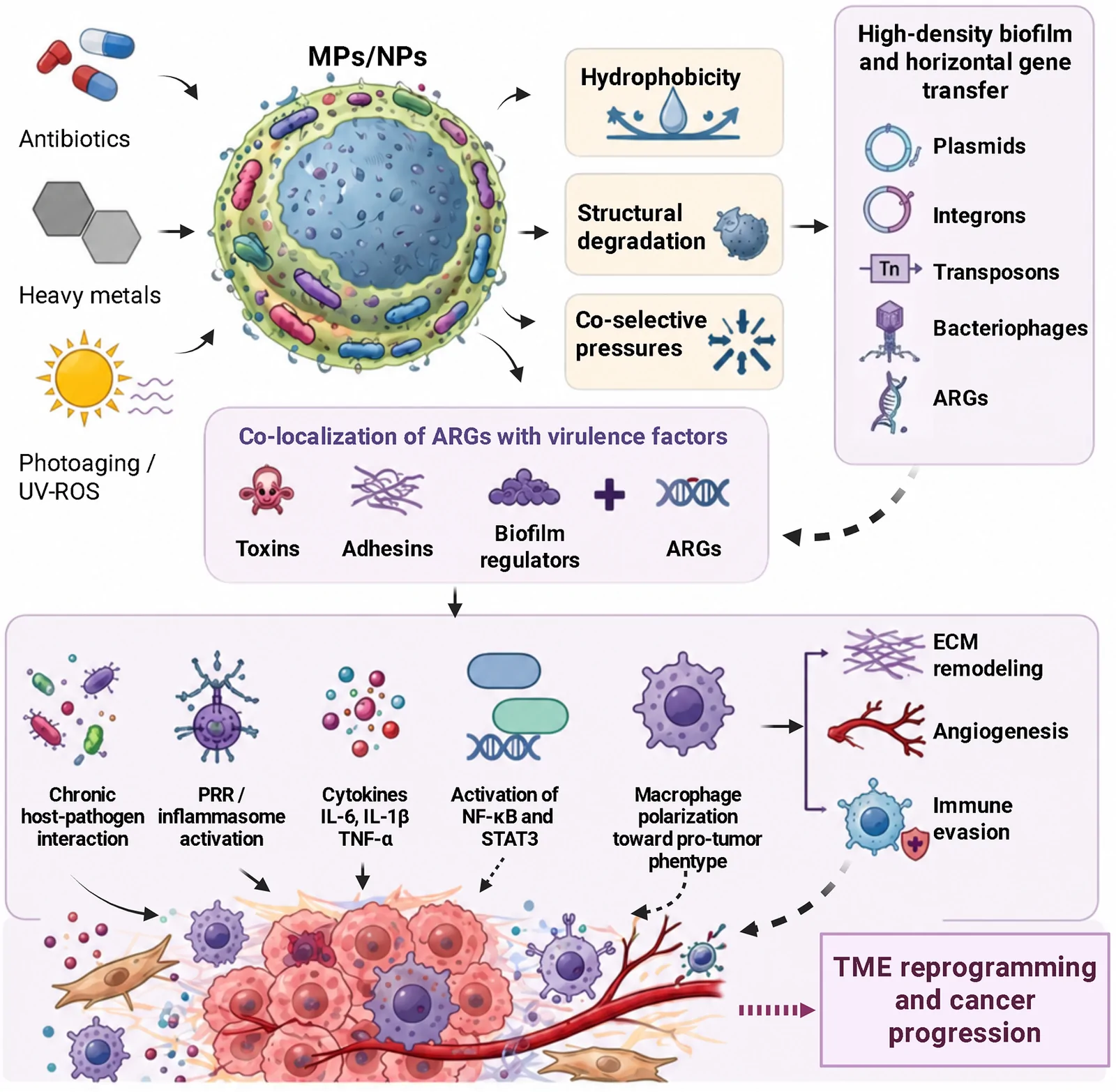
Potential ecological role of MPs/NPs in the environmental resistome: linking ARG dissemination with tumor microenvironment reprogramming. Within the plastisphere, antimicrobial resistance genes (ARGs) may co-localize with virulence determinants, including toxins, adhesins, and biofilm regulators, potentially contributing to microbial persistence and pathogenic potential. These processes may be associated with chronic host–microbe interactions and activation of PRR/inflammasome pathways, cytokine production, and downstream NF-κB and STAT3 signaling in relevant experimental contexts. Potential downstream effects include macrophage polarization toward pro-tumor phenotypes, ECM remodeling, angiogenesis, and immune-evasive responses, although these relationships may be supported primarily by indirect or context-dependent evidence. These pathways may converge on TME reprogramming and cancer progression. Solid arrows indicate mechanisms supported by direct experimental evidence in the reviewed literature, whereas dashed arrows indicate proposed or inferred mechanisms supported primarily by indirect or context-dependent evidence. Overall, the figure presents a hypothesis-generating framework linking plastisphere-associated resistomes, inflammatory signaling, and potential tumor-promoting niche formation. (Created with BioRender.com).

## 7 Limitations

The proposed framework should be interpreted with caution given several limitations in the current evidence base. Although research on the biological effects of MPs/NPs has expanded rapidly, most data derive from *in vitro* and animal models, while direct human evidence remains scarce. Consequently, many mechanistic links discussed here are supported by converging lines of preclinical and indirect evidence rather than by continuous validation across experimental, epidemiological, and clinical settings. Compounding this, studies vary substantially in design, including exposure route, duration, dose, and biological model, and therefore do not consistently capture the complexity of long-term human exposure under environmentally relevant conditions. An additional challenge is the considerable heterogeneity of particles used across studies; most experimental models rely on pristine PS spheres, whereas real-world MPs/NPs are far more diverse in size, morphology, polymer composition, surface chemistry, aging, and associated contaminants or biofilms. Similar variability plagues microbiome research, where differences in host species, baseline microbiota, sequencing approaches, and bioinformatic pipelines often yield inconsistent findings. These limitations underscore the urgent need for methodological standardization, environmentally realistic exposure models, and well-designed longitudinal human studies.

A further conceptual caveat concerns the extrapolation of plastisphere findings to host-associated contexts. Most plastisphere studies have been conducted in aquatic, soil, or wastewater systems, environments where biofilms develop under physicochemical conditions vastly different from those within the human body. Upon ingestion or inhalation, MPs/NPs and their associated microbial consortia encounter a cascade of biological fluids, gastric acid, bile salts, digestive enzymes, mucus, pulmonary surfactant, and immune effectors that may disrupt biofilm integrity, alter microbial viability, and reshape surface-adherent communities. Moreover, the rapid formation of a biomolecular corona upon contact with host fluids can compete with or occlude microbial ligands, modifying the particle’s interfacial properties. Thus, the environmental plastisphere and the biocorona-decorated particle that ultimately interfaces with host tissues should be regarded as biologically and functionally distinct entities. This distinction remains underexplored and represents a critical knowledge gap in assessing the translational relevance of plastisphere-associated microorganisms to human health.

## 8 Final considerations

This review is consistent with a conceptual change in how MPs/NPs are understood in cancer-related biology. Rather than behaving as inert environmental debris, MPs/NPs may emerge as biologically active ecological interfaces that can reshape microbial communities, chemical exposures, and host responses across multiple levels of organization. Through the plastisphere, these particles may concentrate pollutants, virulence determinants, antibiotic resistance traits, and microbial communities, thereby potentially increasing exposure to persistent biological and chemical signals.

Across epithelial barriers, immune compartments, and microbial ecosystems, MPs/NPs may promote a coordinated cascade of barrier disruption, microbial translocation, dysbiosis, chronic inflammation, immune dysregulation, and tumor microenvironment remodeling. These convergent processes suggest a mechanistic basis by which MPs/NPs may function as ecological oncogenic co-drivers, potentially linking the exposome to the microbiome and, ultimately, to cancer-relevant host responses. In this framework, carcinogenesis may be not solely the result of cell-intrinsic mutations, but also of sustained ecological perturbations that could favor pathogenic persistence, inflammatory signaling, immune escape, and tumor-permissive niche formation.

By integrating evidence from environmental microbiology, epithelial biology, immunology, and cancer research, this review addresses its central objective by proposing a unified mechanistic model in which MPs/NPs may act as active mediators of host–microbe–tumor crosstalk. This perspective may expand current paradigms of environmentally driven carcinogenesis and highlights the need for future work using translational experimental platforms, biomarker-guided risk stratification, and surveillance strategies. In summary, these advances will be essential to determine whether plastisphere-associated microbial and chemical exposures constitute not only an emerging ecological concern, but also a clinically relevant dimension of cancer risk and progression.
